# Wellens' Syndrome in the Setting of the 2019 Novel Coronavirus (COVID-19)

**DOI:** 10.7759/cureus.13290

**Published:** 2021-02-11

**Authors:** Karim O Elkholy, Elidona Mirashi, Yury Malyshev, Gregory Charles, Sonu Sahni

**Affiliations:** 1 Internal Medicine, Brookdale University Hospital Medical Center, New York, USA; 2 Internal Medicine, Brookdale University Hospital Medical Center, Brooklyn, USA; 3 Cardiology, Maimonides Medical Center, Brooklyn, USA; 4 Cardiology, Brookdale University Hospital Medical Center, Brooklyn, USA; 5 Research Medicine, New York Institute of Technology College of Osteopathic Medicine, New York, USA; 6 Primary Care, Touro College of Osteopathic Medicine, New York, USA

**Keywords:** coronavirus, novel coronavirus, wellens' syndrome, coronary artery angiogram, electrocardiography, acute coronary syndrome, primary percutaneous coronary intervention (pci), troponin i, covid-19, covid-19-associated acute coronary syndrome

## Abstract

The novel coronavirus, severe acute respiratory syndrome coronavirus 2 (SARS-Cov2), is the causative pathogen of coronavirus disease 2019 (COVID-19), which is primarily described as a respiratory illness. However, a wide array of cardiovascular complications has also been described in the setting of COVID-19. Wellens' syndrome, also regarded as a left anterior descending coronary T-wave syndrome, is an electrocardiography (EKG) pattern that indicates critical proximal left anterior descending (LAD) artery stenosis. It is characterized by deeply inverted T-waves or biphasic T-waves in the anterior precordial chest leads in a patient with unstable angina. Patients typically present with symptoms consistent with acute coronary syndrome. To our knowledge, we present the first case of Wellens' syndrome in a patient with a COVID-19 infection. Furthermore, this case describes stenosis of the left circumflex artery, a variant of the unusual angiographic findings associated with Wellens', as it is usually associated with occlusion of the proximal LAD. The pathophysiology of cardiovascular complications associated with COVID-19 is not well-understood; nevertheless, it was reported that mortality from coronary artery disease (CAD) complications is significantly higher in these patient populations. Healthcare providers should also be aware of identifying Wellens' syndrome, as urgent coronary angiography is superior to stress testing.

## Introduction

The 2019 novel coronavirus (COVID-19) is primarily regarded as a respiratory illness with patients presenting with cough, fever, dyspnea, and other constitutional symptoms, often ending in a diagnosis of acute hypoxic respiratory failure. However, with the increasing number of cases, there is concern about the cardiac manifestations of the disease. Early studies have shown a multitude of cardiac ailments in the setting of the novel coronavirus, including myocardial infarction (MI), arrhythmias, myocarditis, and other myocardial injuries [1-2]. As MI is a legitimate concern in this patient population, clinical predictors would greatly benefit clinicians in management and decision-making. An indicator of left anterior descending (LAD) disease is a phenomenon known as Wellens' syndrome, where specific precordial T-wave changes with ischemic symptoms point towards anterior wall myocardial infarction (MI). Criteria of characteristic electrocardiography (EKG) findings include deeply inverted T-waves in the anterior precordial chest leads (V2-V3) or a characteristic biphasic T-wave. The typical presentation of these patients is that of an acute coronary syndrome (ACS), including chest pain at rest and diaphoresis, but patients may present with vague symptoms or be asymptomatic⁠. With the risk of ACS in the setting of COVID-19, clinicians should be aware of the characteristic EKG findings of Wellens' syndrome. Herein, we present, to the best of our knowledge, the first case of Wellens' syndrome in a patient with COVID-19.

## Case presentation

**Figure 1:**
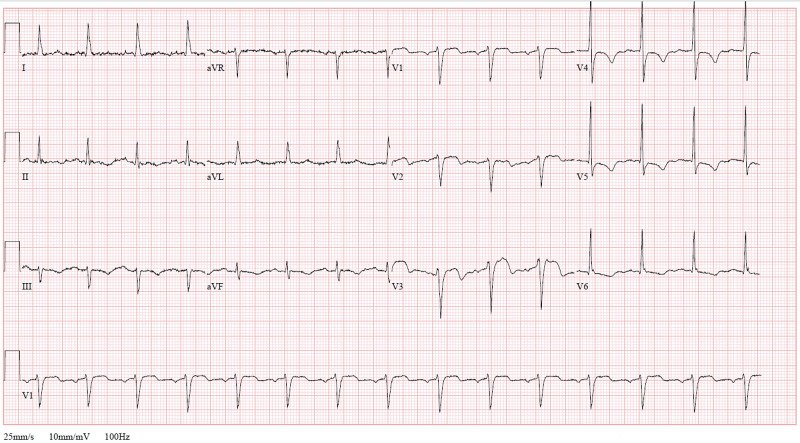
Electrocardiogram showing biphasic T-waves in V2-V3 and T-wave inversions in V4-V5

An 86-year-old African-American male with a prior medical history of coronary artery disease (CAD), status-post percutaneous transluminal coronary angioplasty (PTCA) in an unknown vessel, hypertension, diabetes mellitus, hyperlipidemia, and possible chronic obstructive pulmonary disease presented to the Brookdale University Hospital and Medical Center Emergency Department (ED) with a chief complaint of progressively worsening dyspnea over the past few days. He also reported that he had a feeling of subjective fevers and one episode of non-bloody, non-bilious vomiting at home. Upon further questioning, the patient reported malaise, fatigue, and weakness. However, he denied any chest pain, chills, headache, diaphoresis, cough, sputum production, hemoptysis, or genitourinary symptoms. A review of systems was negative for abdominal pain, constipation, diarrhea, heartburn, or melena. Due to worsening dyspnea, the patient called the emergency medical service (EMS). As per EMS logs, the patient was noted to be saturating at 78% on room air and was placed on 15 liters of oxygen per minute via a non-rebreather mask. Upon arrival to the ED, the patient was alert and oriented, hemodynamically stable with a blood pressure of 131/73 mmHg, heart rate of 106 beats per minute (bpm), a respiratory rate of 20 breaths per minute, oxygen saturation of 100% on oxygen via nasal cannula at 3 liters per minute, and a temperature of 36.9°C (98.4°F). The initial EKG showed sinus tachycardia with nonspecific ST-wave changes. A chest x-ray was performed which showed ground glass consolidations of the bilateral upper and lower lobes. Initial laboratory values are shown in Table 1. For concerns of COVID-19, nasopharyngeal polymerase chain reaction (PCR) testing was performed and revealed reactivity, confirming a diagnosis of COVID-19. The patient was given 2 grams of intravenous magnesium and 125 mg of methylprednisolone. For multilobar pneumonia, ceftriaxone and doxycycline were empirically administered as part of community-acquired pneumonia treatment. The patient remained stable and was weaned from the nasal cannula to room air. Troponin I was noted to trend up to a significant level of 9.980 ng/mL. Repeat EKG showed ST segment depression in the lateral leads, and the patient was started on dual antiplatelet therapy for concerns of non-ST elevation myocardial infarction (NSTEMI). An echocardiogram was performed which showed a left ventricular ejection fraction estimated in the range of 35% to 45% and mildly increased transaortic velocity with the aortic valve area to be 1.5 cm^2^. The troponin I was noted to peak at 14.9 ng/mL. Repeat EKG at that time showed a biphasic T-wave in lead V2-V3, concerning for Wellens' sign, and T-wave inversions in V4-V6 which are shown in Figure 1. Cardiology was consulted and their suggestion was treating the patient as an NSTEMI. Subsequently, the patient was initiated on a full dose of enoxaparin subcutaneous injections at 1 mg/kg every 12 hours and scheduled for cardiac catheterization. COVID-19 testing was noted to come back positive and the patient continued on antibiotics and vitamin supplementation with vitamin C and zinc.

**Table 1:** Clinical Laboratory Values at Presentation and Post-PCI CRP: C-reactive protein; DDU: D-dimer unit; P-BNP: pro-brain natriuretic peptide; PCI: percutaneous coronary intervention; WBC: white blood count

Laboratory tests	Presentation (Day 1)	Post-PCI (Day 3)	Reference normal values
Hemoglobin	10 g/dL	10.2 g/dL	12.9 - 16.7 g/dL
WBC	6.6 10x3/uL	6.4 10x3/uL	4.1 - 10.1 10x3/uL
Absolute lymphocyte count	0.7 10x3/uL	0.9 10x3/uL	1.1 - 2.9 10x3/uL
Troponin I	0.282 ng/mL	6.49 ng/mL	0.012 - 0.034 ng/mL
Creatinine	0.91 mg/dL	1.06 mg/dL	0.66 - 1.25 mg/dL
Blood urea nitrogen	16 mg/dL	33 mg/dL	9/20 mg/dL
Sodium	135 mEq/L	136 mEq/L	133 - 145 mEq/L
Potassium	5.2 mEq/L	4.5 mEq/L	3.5 - 5.1 mEq/L
Magnesium	2.2 mg/dL	2.1 mg/dL	1.6 - 2.3 mg/dL
Lactate dehydrogenase	690 IU/L	891 IU/L	313 - 618 IU/L
Ferritin	63.7 ng/mL	74.5 ng/mL	17.9 - 464 ng/mL
CRP	1.3 mg/dL	1.6 mg/dL	0.5 - 1 mg/dl
Erythrocyte sedimentation rate	58 mm	-	0 - 20 mm
D-dimer	451 n/mL DDU	261 ng/mL DDU	0 - 230 ng/mL DDU
P-BNP	5,910 pg/mL	-	11.1 - 450 pg/mL

**Figure 2:**
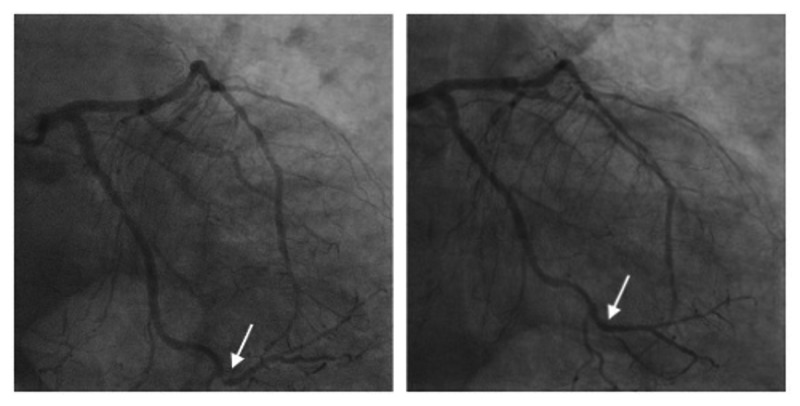
Angiogram images before and after the intervention Left: angiogram image showing disease in the distal left circumflex (notice discontinuation in the contrast material; white arrow on the left); Right: angiogram image showing distal left circumflex following stent placement (notice restoration of blood flow; white arrow on the right)

Cardiac catheterization was performed via the right radial artery and revealed significant triple vessel disease. Chronic total occlusion of the right coronary artery (RCA), severe disease of the first diagonal, and severe stenosis of the distal obtuse marginal 1 (OM1) were observed. Nevertheless, the remainder of the angiogram revealed normal coronaries. Balloon angioplasty with stent placement was performed on the 95% lesion in the distal circumflex. Following the intervention, there was a 0% residual stenosis and no dissection present as shown in Figure 2. Post-procedure, the patient continued taking aspirin and ticagrelor as part of a dual antiplatelet regimen. Goal-directed medical therapy with a beta-blocker and high-intensity statin therapy for heart failure with reduced ejection fraction was initiated and the patient was discharged with outpatient follow-up.

## Discussion

To our knowledge, this is the first case of Wellens' syndrome in a patient with COVID-19. Wellens' syndrome was first described in 1982 by de Zwaan et al. after recognizing specific EKG changes of patients with a high risk for development of an extensive anterior wall MI [3]. Criteria for the diagnosis of Wellens' syndrome include 1) deeply inverted or biphasic T-waves in V2 and V3, in addition to absent precordial Q-waves, 2) normal precordial R-wave progression, 3) insignificant ST elevation usually < 1 mm, and 4) normal or minimally elevated cardiac serum enzymes [4]⁠. They observed that 16% of the 145 patients who were admitted because of unstable angina had these characteristic EKG findings, indicating that this finding was not rare. Since then, patients with unstable angina and EKG findings suggestive of Wellens' sign are recommended to undergo urgent coronary angiography and revascularization of the occluded LAD coronary [3]. In a larger study by de Zwaan et al., it was observed that all of the 180 out of the 1,260 patients admitted for unstable angina with these characteristic EKG changes had stenosis of the LAD, ranging from 50% to complete obstruction [5]. While Wellens' syndrome, alternatively called left anterior descending coronary T-wave syndrome, has been usually described with LAD occlusion, a recent report by Ajibawo et al. described Wellens' sign with proximal circumflex artery occlusion [6]. Our case is another example of circumflex artery obstruction presenting with Wellens' sign on EKG. The relationship between COVID-19 and its acute vascular events, including acute myocardial infarction, has been noted. The incidence of myocardial injury among COVID-19 patients was previously reported as 7.2% - 20% in two different studies [1-2]. A recently published case series described 18 patients with COVID-19 who presented with ST segment elevation and had significantly elevated inpatient hospital mortality (72%). Fourteen patients had only focal ST elevation (78%), while four patients had diffuse ST elevations (22%) [7]. COVID-19 has been associated with other cardiovascular complications, including new onset or exacerbation of heart failure, myocarditis, or other acute myocardial injuries, such as myocardial infarction. However, the pathophysiology is not yet well-understood. Data on COVID-19 complicated by heart failure is limited. However, a retrospective study of 799 patients hospitalized with COVID-19 in Wuhan described 49% of patients who died and 3% of patients who survived had heart failure as a complication [8]. In addition, there have been reports of myocarditis in patients with COVID-19 [9-10]. This case reports a unique finding in Wellens' syndrome, as to our knowledge, this is the first case of Wellens' syndrome described in a COVID-19 patient. Furthermore, this case describes a variant of the usual angiographic findings associated with Wellens' since it is usually associated with occlusion of proximal LAD and this case describes stenosis of the left circumflex artery.

## Conclusions

Although our case might represent atypical coronary angiogram findings in EKG changes consistent with Wellens' sign that might be related to the patient's COVID-19 status, physicians should be aware of the characteristic EKG findings associated with Wellens’ syndrome. Identification of Wellens’ sign on the EKG is crucial, as the need for an urgent angiogram is superior to cardiac stress testing due to Wellens’ usual association with underlying coronary artery lesions. Moreover, healthcare providers should be aware of COVID-19-associated CAD complications. While CAD in COVID-19 patients is linked to worse outcomes, further reports might help us understand the pathophysiology of the cardiovascular complications of this disease.
